# Co-Circulation of Multiple Coronavirus Genera and Subgenera during an Epizootic of Lethal Respiratory Disease in Newborn Alpacas (*Vicugna pacos*) in Peru: First Report of Bat-like Coronaviruses in Alpacas

**DOI:** 10.3390/ani13182983

**Published:** 2023-09-21

**Authors:** Luis Llanco, Karubya Retamozo, Noriko Oviedo, Alberto Manchego, César Lázaro, Dennis A. Navarro-Mamani, Norma Santos, Miguel Rojas

**Affiliations:** 1 Escuela de Medicina Humana, Universidad Privada San Juan Bautista, Apartado, Chincha 15067, Peru; luis.llanco@uspsjb.edu.pe; 2 Laboratório de Inmunología, Facultad de Medicina Veterinaria, Universidad Nacional Mayor de San Marcos, Apartado, Lima 03-5137, Peru; karu.3.93@gmail.com (K.R.); yahaira.oviedo@unmsm.edu.pe (N.O.); amanchegos@unmsm.edu.pe (A.M.); 3 Laboratório de Farmacología y Toxicología Veterinaria, Facultad de Medicina Veterinaria, Universidad Nacional Mayor de San Marcos, Apartado, Lima 03-5137, Peru; clazarod@unmsm.edu.pe; 4 Laboratório de Virología, Facultad de Medicina Veterinaria, Universidad Nacional Mayor de San Marcos, Apartado, Lima 03-5137, Peru; dnavarrom@unmsm.edu.pe; 5 Instituto de Microbiologia Paulo de Góes, Universidade Federal do Rio de Janeiro, Rio de Janeiro 21941-902, RJ, Brazil; nsantos@micro.ufrj.br

**Keywords:** alpaca, BatCoV, *β*-CoV, Embecovirus, *α*-CoV, decacovirus, mortality, Peru

## Abstract

**Simple Summary:**

Alpacas (*Vicugna pacos*) and llamas (*Lama glama*) constitute the most significant livestock wealth of the Andean populations of South America. Infectious diseases, particularly respiratory and enteric infections, cause high morbidity and mortality in offspring and adult animals. In this study, we demonstrated that multiple variants of the coronavirus co-circulated among Peruvian alpacas. We also demonstrated that some of these variants bear similarities to coronavirus strains detected in bats. For a better understanding of the infections that afflict these animals, continuous surveillance is needed to identify the emergence of new genotypes and viral variants that are potentially pathogenic to alpacas and humans. Future studies should include the sequencing of genes encoding CoV spike proteins and host receptors to confirm interspecies transmission.

**Abstract:**

Coronaviruses (CoVs) infect a wide range of hosts, including humans, domestic animals, and wildlife, typically causing mild-to-severe respiratory or enteric disease. The main objective of this study was to identify CoV genera and subgenera detected in Peruvian alpacas. Lung lavage specimens were collected from 32 animals aged 1 to 6 weeks. CoVs were identified by using RT-PCR to amplify a pan-CoV conserved region of the RNA-dependent RNA polymerase-encoding gene. A nested PCR was performed to identify *β*-CoVs. Then, *β*-CoV-positive samples were subjected to RT-PCR using specific primers to identify the *Embecovirus* subgenus. Out of 32 analyzed samples, 30 (93.8%) tested positive for at least one CoV genus. *β*-, *α*-, or unclassified CoVs were identified in 24 (80%), 1 (3.3%), and 1 (3.3%) of the positive samples, respectively. A CoV genus could not be identified in two (6.7%) samples. A mixture of different CoV genera was detected in two (6.7%) samples: one was co-infected with *β*- and *α*-CoVs, and the other contained a β- and an unclassified CoV. A sequence analysis of the amplicons generated by the PCR identified 17 *β*-CoV strains belonging to the subgenus *Embecovirus* and two *α*-CoV strains belonging to *Decacovirus*. A phylogenetic analysis of two strains revealed a relationship with an unclassified *Megaderma* BatCoV strain. A subgenus could not be identified in nine *β*-CoV samples. Our data show a high prevalence and a high genetic diversity of CoV genera and subgenera that infect alpacas, in which the *β*-CoV subgenus *Embecovirus* predominated. Our data also suggest a new role for bats in the dissemination and transmission of uncommon CoVs to alpacas raised in rural Peru.

## 1. Introduction

Raising alpacas (*Vicugna pacos*) and llamas (*Lama glama*) is the main economic activity of the Andean populations of southern Peru [1]. The Peruvian alpaca herd represents 85% of the world’s population of these animals. Breeding is distributed primarily (77%) in the southern highlands in the departments of Puno and Cuzco [2,3,4]. Most alpaca farms are small (50–100 animals) and conduct extensive breeding with inappropriate livestock management [2,3,5]. 

Neonatal mortality in alpacas can reach up to 30% [2], which is primarily due to respiratory and enteric infections following the failure of passive transfer colostrum antibodies [6,7]. Co-circulating viruses and bacteria have been implicated in outbreaks that feature co-infections due to highly lethal pathogens such as *Streptococcus pneumoniae*, *Mannheimia haemolytica*, and *Pasteurella multocida* [8]; coronavirus (CoV), mammalian orthoreovirus, and rotavirus *A* [9,10,11,12]; CoV and *Salmonella* spp. [13]; parainfluenza virus type 3, bovine respiratory syncytial virus, *Pasteurella multocida*, and *Mannheimia haemolytica* [14,15]. 

CoVs infect a wide range of hosts, including humans, domestic animals, and wildlife, typically causing mild-to-severe respiratory or enteric disease [16]. This family of viruses, with a positive-sense single-stranded RNA genome, exhibits a high genetic diversity and is classified into four genera: *Alphacoronavirus* (*α*-CoV), *Betacoronavirus* (*β*-CoV), *Gammacoronavirus* (*γ*-CoV), and *Deltacoronavirus* (*δ*-CoV) [17]. In general, *α*-CoV and *β*-CoV infect mammalian hosts, while *γ*-CoV and *δ*-CoV infect birds, although some of them also infect mammals. It has been suggested that bats are the genetic source of α-CoV and *β*-CoV, while birds are the genetic source of *γ*-CoV and *δ*-CoV. Bat CoVs, in addition to infecting several bat species, cross the interspecies barrier infecting other mammals, including humans. Similarly, CoVs from birds have acquired the ability to infect a variety of bird species and, occasionally, some mammalian species, such as whales and pigs [18]. Each genus is further divided into subgenera and species, characterized by great genetic diversity resulting from the high frequency of homologous recombination and the accumulation of point mutations, which confer the ability to cross species barriers [17,19]. Recurrent events of interspecies transmission represent the potential for accelerating viral evolution and, consequently, the possibility of the emergence of new viral strains. The cohabitation of birds and mammals in domestic and wild environments, as well as proximity to humans, may offer the possibility of crossing the interspecies barrier and eventually lead to the emergence of new variants capable of adapting to new hosts, including humans, as observed in SARS-CoV-1, SARS-CoV-2, and MERS-CoV [17,18,20]. Based on phylogenetic analyses, it appears that all human coronaviruses have animal origins: SARS-CoV, MERS-CoV, HCoV-NL63, and HCoV-229E are considered to have originated in bats; HCoV-OC43 and HKU1 likely originated in rodents [17]. Domestic animals may have played important roles as intermediate hosts that allow for the transmission of the virus from natural hosts to humans. The camelids were likely intermediate hosts of HCoV-229E [21,22], and HCoV-OC43 likely evolved from ancestral BCoV strains that crossed the interspecies barrier and established infections in humans [23,24].

SARSCoV-1, SARSCoV-2, and MERS-CoV are examples of viruses that emerged in the human population after spillover events, likely from an animal reservoir, with devastating effects on public health; these viruses are classified as *β*-CoVs that originated from bats, which are transmitted to humans through intermediate hosts such as civets, pangolins, and old-world camelids, respectively [17,20]. Natural and experimental infections by SARS-CoV-2 have been described in a wide variety of animal hosts. Ferrets and cats were found to be highly susceptible to the virus, while dogs are less susceptible, and chickens, ducks, and pigs have shown lower susceptibility [25,26]. The free-ranging white-tailed deer has also been shown to be highly susceptible to SARS-CoV-2 virus infection and capable of sustaining transmission in nature [27]. Serological evidence has demonstrated the ability of SARS-CoV-2 to naturally infect small ruminants such as cattle, sheep, goats, and dromedary camels [28,29]. A SARS-CoV-2 spillback transmission from humans to animals has been suggested [30,31,32], raising the possibility of SARS-CoV-2 amphixenosis.

Bovine CoVs (BCoVs) that replicate in the intestine, infect the upper and lower respiratory systems, and are commonly associated with enteric disease in cattle. Domestic (goats, sheep, water buffalos, dromedary camels, alpacas, and llamas) and wild ruminants (reindeer, elk, sambar deer, sika deer, musk oxen, wisents, wood bison, waterbucks, sitatungas, stable antelopes, nyalas, giraffes, and Himalayan tahrs) are infected by CoV strains that share biological, antigenic, and genetic similarities with BCoVs (called bovine-like CoVs or BCoV-likes) [16,33]. BCoV-likes have also been detected in other species such as Indonesian tapirs (*Acrocodia indica*), an ungulate but non-ruminant species, with dysentery [34]; dogs with respiratory disease [35]; and humans with diarrhea [36], showing the ability of BCoV-likes to adapt to new hosts.

CoVs detected in Peruvian and other South American alpacas have been associated with enteritis caused by bovine-like coronavirus strains [10,11,37,38], identified as a *β*-CoV of the *Embecovirus* subgenus. In North America, viruses belonging to the *β*-CoV genus (subgenus *Embecovirus*) and *α*-CoV (subgenus *Duvinacovirus*) have been reported in alpacas with enteric and respiratory diseases, respectively [13,39,40].

This study aimed to identify the genera and subgenera of CoVs present in bronchial lavage samples obtained from newborn alpacas in Cuzco.

## 2. Materials and Methods

### 2.1. Sampling

Lung lavage specimens were collected from newborn alpacas (n = 32) between one and six weeks of age from the rural community of Silly, located in the District of Marangani, Province of Canchis, Department of Cuzco, Peru (14°21′12″ S, 71°10′17″ W, 3800 masl) during the birthing season of 2012. Samples were collected directly from lungs during necropsy and stored at −70 °C until processing at the Laboratory of Veterinary Virology and Immunology at the Facultad de Medicina Veterinaria of the Universidad Nacional Mayor de San Marcos (FMV-UNMSM), Lima, Peru. 

### 2.2. Viral Detection and Identification

Viral RNA was extracted from the lung lavage using TRIzol™ LS Reagent (Thermo Fisher Scientific, Waltham, MA, USA) according to the manufacturer’s instructions. Samples were tested for the presence of CoVs via reverse-transcription PCR amplification (RT-PCR) and nested PCR using specific primers targeting a 251 bp fragment of the RNA-dependent RNA polymerase gene (RdRp), which is conserved across all CoVs (Table 1). Briefly, the viral RNA was subjected to one reverse-transcription cycle consisting of 5 min at 25 °C followed by 45 min at 42 °C and one step of 2 min at 95 °C followed by PCR cycles as described elsewhere [41]. The generated amplicons were submitted to nested PCR using specific primers for β-CoV to generate a 227 bp from RpRd [9]. β-CoV-positive samples were further analyzed to identify subgenus *Embecovirus* using nested PCR. The PCR conditions were as previously described by Brandão et al., 2004 [42] (Table 1). PCR products were separated with 1.5% (*w*/*v*) agarose gel electrophoresis, stained with ethidium bromide, and visualized under UV light. A 100 bp DNA ladder (Promega, Madison, WI, USA) was used to determine molecular size.

To validate the PCR assays, positive controls were used for each of the four CoV genera (*α*-CoV, *β*-CoV, *γ*-CoV, and *δ*-CoV), which included CoVs isolated from pigs, chickens, and alpacas and belonging to the collection of the Laboratory of Veterinary Virology and Immunology of the FMV-UNMSM. The alpaca CoV strains used as positive controls were AlpCoV-SA44 and AlpCoV-HN (GenBank accession numbers KX266949 and KX266944, respectively), both belonging to *β*-CoV; subgenus, *Embecovirus*; species, bovine-like CoV.

### 2.3. CoV Characterization via Phylogenetic Analysis of Partial Sequences of the RdRp Gene

Amplified genomic segments were sequenced by Macrogen Inc. (Seoul, Republic of Korea). Overlapping sequences were assembled and edited using SeqMan, EditSeq, and MegAlign in the Lasergene software package (Version 7.0, DNASTAR, Madison, WI, USA). Phylogenetic analysis was performed with the MEGAX software [43,44]. Dendrograms were constructed using the maximum likelihood method based on the Hasegawa–Kishino–Yano model [43]. Statistical significance was estimated via bootstrap analysis with 1000 pseudoreplicates. Sequences were compared with reference CoV strains obtained from GenBank (https://www.ncbi.nlm.nih.gov/nucleotide/, accessed on 1 August 2023). Sequences generated in this study were deposited in GenBank under accession numbers OQ845932–OQ845939.

## 3. Results

### 3.1. Detection and Identification of CoV Genera and Subgenera

**Figure 1:**
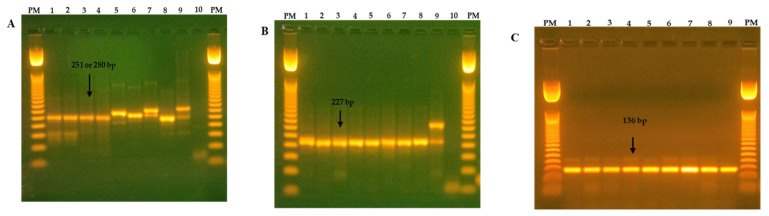
Ethidium bromide-stained 1.5% agarose gel showing the amplified products of a pancoronavirus RT-PCR (251 or 280 bp) from a nested-PCR of betacoronavirus (227 bp) and an Embecovirus nested-PCR (136 bp). (**A**) RT-PCR pancoronavirus (CoV, RdRp gene): PM line, 50 bp of DNA marker; lines from 1 to 9; lung lavage samples; lane 10, negative control. (**B**) Nested-PCR (*β*-CoV, RdRp gene): PM line, 50 bp of DNA marker; lines from 1 to 9; lung lavage samples; lane 10, negative control. (**C**) Nested-PCR (Embecovirus, RdRp gene): PM line, 50 bp of marker DNA; lines from 1 to 9; lung lavage samples. Note: Pancoronavirus products in alpacas can vary in size from 251 to 280 bp; in some cases, both bands are present; PM = molecular weight.

**Figure 2:**
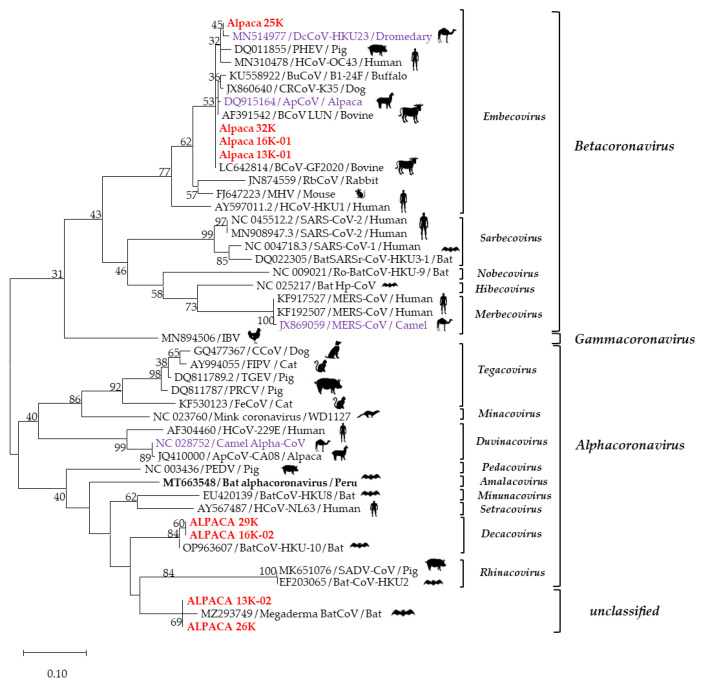
Phylogenetic relationships of coronaviruses based on a 251 bp fragment of RdRp gene. Sequences in red are of our study, and sequences in purple are of different CoV species reported in other camelids. This analysis involved 46 nucleotide sequences identified with GenBank accession numbers. Dendrograms were constructed using the maximum likelihood method; the distances were corrected with the Hasegawa–Kishino–Yano model. Statistical support was provided by bootstrapping 1000 pseudoreplicates. Bootstrap values >75% are given at branch nodes. The distance scale reflects substitutions/sites.

Of the 32 lavage samples, 30 (93.8%) tested positive for at least one CoV genus. Twenty-six samples were positive for *β*- (24; 80%), *α*- (1; 3.3%), or unclassified (1; 3.3%) CoVs. In two (6.7%) RT-PCR-positive samples, the CoV genus could not be identified with either nested PCR or sequencing (Figure 1; Table 2). The detection of multiple CoV genera was observed in two (6.7%) samples. One sample contained a mixture of *β*- and *α*-CoVs, and the other contained *β*-CoV and an unclassified CoV (Table 2). The identification of the subgenera of the detected strains showed that 17 strains of *β*-CoV belonged to the subgenus *Embecovirus*; two *α*-CoV strains belonged to the genus *Decacovirus*. Two strains showed phylogenetic relationships to an unclassified *Megaderma* bat-CoV strain. Subgenera could not be identified for nine *β*-CoV strains (Figure 2; Table 2). 

### 3.2. Phylogenetic Analysis of the RdRp Gene

Of the eighteen products selected for sequencing the partial *RdRp* gene (251 bp), eight were successfully sequenced: 251 bp of four samples (Alp 13K-01, Alp 16K-01, Alp 25K, and Alp 32K) and 112 bp of four other samples (Alp 13K-02, Alp 16K-02, Alp 26K, and Alp 29K). A phylogenetic analysis grouped the sequences into three distinct clusters, each belonging to a different genus: *β*-CoV (EmbeCoV) or *α*-CoV (DecaCoV). Two sequences were grouped with a bat coronavirus (BatCoV) strain not yet classified by the ICTV (Figure 2).

Strains 13K-01, 16K-01, 25K, and 32K shared a 98.8%-to-100% nucleotide identity. When compared with CoV reference sequences, they showed a phylogenetic relationship closer to strains of the subgenus *Embecovirus*, with nucleotide identities in a range of 80.9% to 100%, phylogenetically closer to CoVs detected in bovines (BCoV) (LC642814/GF2020 and AF391542 /LUN), alpaca (ApCoV) (DQ915164), and dromedary (DcCoV) (MN514977), with nucleotide identities of 98.8–100%, 98.4–99.6%, 99.2–98%, and 99.2–98.8%, respectively. On the other hand, these strains showed 65.4%, 69.9%, 63.2%, and 58.1% nucleotide identities with reference strains of the *β*-CoV subgenera *Sarbecovirus*, *Nobecovirus*, *Hibecovirus*, and *Merbecovirus*, respectively. 

The amplicon positives yielded by pancoronavirus and panbetacoronavirus RT-PCRs that were negative for the *Embecovirus* subgenus could not be sequenced correctly with the Sanger method because they presented excessive noise during chromatography.

Sequences 29K and 16K-02 were 100% identical and showed a phylogenetic relationship to the BatCoV/HKU strain (OP963607; 99.1% nucleotide identity), which is classified as an *α*-CoV (DecaCoV). Sequences 13K-02 and 26K were identical and phylogenetically closest (98.1% nucleotide identity) to the *Megaderma* CoV strain (MZ293749/Bat-CoV), which is not yet classified in any coronavirus genus. The phylogenetic distance obtained by aligning the 29K strain with the 32K, 25K, and 26K strains resulted in nucleotide identities of 66.9%, 64.6%, and 86.5%, respectively. Co-infections were confirmed by sequencing two samples: 16K (*β*-CoV + *α*-CoV) and 13K (*β*-CoV + unclassified CoV) (Figure 2, Table 2).

## 4. Discussion

Our results demonstrated the presence of CoV in 93.8% (32/30) of lung lavage samples from newborn alpacas from Cuzco, Peru. Other reports have described outbreaks of diarrhea in alpacas from rural communities located in the Departments of Cuzco and Junín, presenting rates of 87.5% (70/80) and 53.3% (32/60), respectively [9,45]. 

The *β*-CoV genus was the most prevalent (86.7%; 26/30), consistent with the data presented by Castilla et al. [9], which detected *β*-CoVs associated with diarrhea in neonatal alpacas (94.3%; 66/70). However, in that outbreak, the *Embecovirus* subgenus was identified in only 22.9% (16/70) of samples. Furthermore, the subgenus of 71.4% (50/70) of the *β*-CoV strains could not be identified [9]. On the other hand, in our study, Embecoviruses represented the majority (65%; 17/26) of strains, and yet, subgenus identification was not possible for 35% (9/26) of the strains. These data reveal the co-circulation of distinct viral variants in the studied region. The variation in the prevalence of Embecoviruses could be multifactorial. Although both studies were conducted in the same geographic area, in the rural community of Silly during the alpaca birthing season (January and February), clinical samples were obtained from different anatomic sites (fecal versus lung lavage specimens), which could suggest differential tissue tropisms. On the other hand, the year during which the samples were obtained differed between studies. Castilla et al. analyzed samples obtained in 2015, while those analyzed in this study were collected in 2012. Given the CoV capacity for rapid evolution [17], *β*-CoV strains belonging to one or more subgenera, distinct from *Embecovirus*, that were already circulating in alpaca herds in 2012 may have adapted to this host, becoming more prevalent over the years and subsequently representing more than 70% of circulating strains in 2015. Therefore, it is essential to monitor these viruses continually to better understand their dynamics in the environment.

In the present study, we report a minor percentage of CoV-positive samples in which a genus could not be identified, similar to previous findings [9]. Our attempts to isolate these viruses in Vero cell cultures (African green monkey kidney cells) were unsuccessful, perhaps because of the long storage period of the samples. These results indicate that studies using next-generation sequencing techniques and continuous molecular surveillance are necessary to better understand the epidemiology and genetic diversity of CoVs in Peruvian alpacas.

The sequencing of four strains (Alp16K-01, 13K-01, 25K, and 32K), classified as *β*-CoVs/*Embecoviruses*, showed a close relationship with BCoVs, in agreement with previous studies suggesting that either the alpaca and BCoVs arose from a common ancestor or that BCoVs are continuously transmitted to alpacas [11,38,39]. Although South American camelids have been in contact with cattle for about five centuries, recent reports of disease associated with BCoVs may be due to the emergence of new CoV variants that are pathogenic to both bovines and camelids [13,39]. These results can also be explained by geographic and climatologic conditions, where different animal species (pigs, cows, llamas, horses, etc.) coexist and graze in proximity [45].

Two strains (16K-02 and 29K) were classified as *α*-CoVs/*Decacoviruses* closely related to a bat CoV (bat CoV/HKU; OP963607). This is not the first report of *α*-CoV infections in alpacas; however, the previously reported subgenus was *Duvinacovirus*, closely related to human coronavirus 229E (HCoV-229E), one of the most prevalent common cold coronaviruses in humans [40]. Subsequent genetic studies suggest that the progenitor of HCoV-229E is an African bat CoV and that camelids were probably the first intermediate hosts that facilitated transmission to humans [17,21,46,47]. This suggests that bats located in the region under study may be transmitting various CoV species with the capacity to adapt and become pathogenic to new host species. To the best of our knowledge, our study is the first to report *α*-CoV infections in Peruvian alpacas.

A genus was not identified in a pair of samples (13K-02 and 26K); these sequences are genetically very similar to a bat CoV strain (MZ293749/*Megaderma* CoV), which remains unclassified. This confirms both the great diversity of this viral family and the role of bats as an important reservoir of these and other unclassified viruses [48,49,50].

An important observation in this study was the detection of multiple CoV genera in two samples confirmed through the sequencing of a fragment of *RdRp*. Genomic recombination has been shown to be an important factor in CoV evolution but requires that different viral strains infect the same host and the same cells simultaneously [17,51,52]. In fact, So et al. demonstrated that a strain of MERS-CoV that infects African dromedary camels (DcCoV-HKU23; subgenus, *Embecovirus*) was a recombinant of bovine, rabbit (RbCoVHKU14), and rodent CoVs [51]. We suggest that CoVs circulating in alpacas are in active recombination, which favors the evolution of *β*-, *α*-, and other CoV genera not yet detected or classified. 

**Figure 3:**
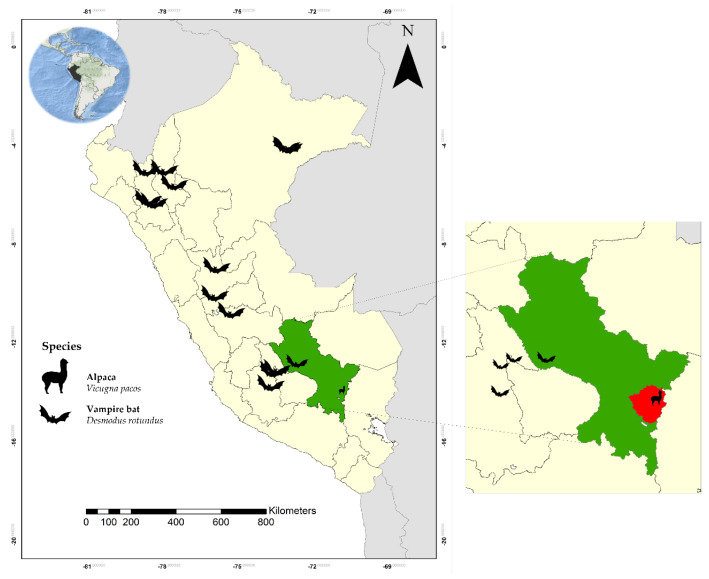
Location of Peruvian bat sampling sites in a study by Bergner [54]. In green, the Department of Cuzco, Peru, and in red, the sampling site of the alpacas for our study.

Because bats are widely distributed in Peru, they could be the source of the CoV strains in samples 13K-02, 16K-02, 26K, and 29K. For example, *Desmodus rotundus*, a hematophagous bat observed at up to 3680 masl, inhabits the South Andean region (Cusco); alpacas also share this geographic space (Figure 3) [53,54]. The invasion of their habitat, a hematophagous diet, migration due to climate change, and proximity to alpacas may favor interspecies spillover. The transmission of enzootic bat viruses to domestic animals was reported previously. The HKU2-related BatCoV caused a large-scale epizootic of enteric disease in China, resulting in the deaths of more than 24,000 piglets. This outbreak demonstrated that the spillover of a BatCoV can cause severe disease in livestock [17,55]. Bat species are a common origin for most CoVs affecting humans [21]. The intermediate hosts for these CoVs usually belong to other mammalian species [46]. The general ecological separation between bats and humans implies the need for other mammals to act as link hosts between bats and humans [56].

Bergner et al. [54], using metagenomics, characterized viral communities from salivary and fecal specimens obtained from wild bats captured in caves and trees located in high Andean and jungle areas of the Departments of Cusco, Ayacucho, and Amazonas in Peru in a period from 2015 to 2016 (Figure 3) and demonstrated that a subset of CoVs that infect neonatal alpacas—specifically, *α*-CoV (*Decacovirus* subgenus in our study) and other CoVs of a currently unclassified genus—are products of interspecies transmission between bats and alpacas. This can also be explained because the area where these *α*-CoVs were identified is endemic to bats [53,54] and close to the collection area of our study. 

## 5. Conclusions

Our data showed the high prevalence and the high genetic diversity of CoV genera and subgenera detected in alpacas, in which the *β*-CoV subgenus *Embecovirus* predominated. Our data also demonstrated the genetic similarity between strains of CoVs circulating in Peruvian alpacas and bats. Given the high transmissibility and the zoonotic nature of coronaviruses, continuous surveillance is necessary to identify the emergence of new viral genotypes and variants potentially pathogenic to alpacas and humans. Future studies should include the sequencing of genes encoding CoV spike proteins and host receptors to confirm interspecies transmission.

## Figures and Tables

**Table 1:** Primers used in the RT-PCR and nested PCR assay for CoV detection †.

Virus	Gene	Assay	Primer *	Primer Sequence 5′ → 3′	Position	Product Size (bp)	Reference
All CoV	RdRd ^+^	RT-PCR	Cor-FW	ACWCARHTVAAYYTNAARTAYGC	14,922–14,944	251	[41]
			Cor-RV	TCRCAYTTDGGRTARTCCCA	15,153–15,172		
*β-CoV*	RdRd	Nested PCR	Beta.CoV.F	ATTAGTGCWAAGAATAGAGCYCGCAC	14,946–14,971	227	[9]
			Beta.CoV.R	TCACAYTTWGGRTARTCCCADCCCA	15,148–15,172		
*Embecovirus*	RdRd	Nested PCR	CV2U.F	TACTATGACTGGCAGAATGTTTCA	14,996–15,019	136	[42]
			CV2L.R	AACATCTTTAATAAGGCGRCGTAA	15,108–15,131		

† CoV = coronavirus; ^+^ RdRp = RNA-dependent RNA polymerase. * The primers’ positions were determined based on the reference CoV strain DQ915164.

**Table 2:** Identification of genera and subgenera of coronaviruses in samples from lung lavages of Peruvian alpacas killed in a respiratory outbreak.

Infection Type	Genus	Subgenus	Nº Positive Samples
Single detection	*Betacoronavirus* (*β-CoV*)	*Embecovirus* (EmbeCoV)	15
		Not identified	9
	*Alphacoronavirus* (*α-CoV*)	*Decacovirus* (DecaCoV)	1
	Unclassified (Megaderma Bat-CoV-like)	Unclassified (Megaderma Bat-CoV-like)	1
	Not identified	Not identified	2
Multiple detection	*β-CoV* + *α-CoV*	EmbeCoV + DecaCoV	1
	*β-CoV* + Unclassified (Megaderma Bat-CoV-like)	EmbeCoV + unclassified	1
Total			30

*β-CoV* = *Betacoronavirus*; EmbeCoV = *Embecovirus*; *α-CoV* = *Alphacoronavirus*; DecaCoV= *Decacovirus*; Unclassified = CoV without a determined genus (ICTV, 2023); not identified = products of 251 bp without success in sequencing.

## Data Availability

Not applicable.
